# Production, statistical optimization, and functional characterization of alkali stable pectate lyase of *Paenibacillus lactis* PKC5 for use in juice clarification

**DOI:** 10.1038/s41598-022-11022-0

**Published:** 2022-05-09

**Authors:** Priyanka Sheladiya, Chintan Kapadia, Vimal Prajapati, Hesham Ali El Enshasy, Roslinda Abd Malek, Najat Marraiki, Nouf S. S. Zaghloul, R. Z. Sayyed

**Affiliations:** 1 Department of Plant Molecular Biology and Biotechnology, ASPEE College of Horticulture and Forestry, Navsari Agricultural University, Navsari, 396450 India; 2 ASPEE SHAKILAM Biotechnology Institute, Navsari Agricultural University, Ghod Dod Road, Athwa Farm, Surat, 395 007 India; 3 Institute of Bioproduct Development, Universiti Teknologi Malaysia (UTM), 81310 Skudai, Johor Bahru, Malaysia; 4 School of Chemical and Energy Engineering, Faculty of Engineering, Universiti Teknologi Malaysia (UTM), 81310 Skudai, Johor Bahru, Malaysia; 5 City of Scientific Research and Technology Applications, New Burg Al Arab 21934, Alexandria, Egypt; 6 Department of Botany and Microbiology, College of Science, King Saud University, P.O. 8455, Riyadh, 11451 Saudi Arabia; 7 Bristol Centre for Functional Nanomaterials, HH Wills Physics Laboratory, Tyndall Avenue, University of Bristol, Bristol, BS8 1FD UK; 8 Department of Microbiology, PSGVP Mandals Shri S I Patil Arts, G B Patel Science, and STKVS Commerce College, Shahada, 425409 India

**Keywords:** Biotechnology, Microbiology

## Abstract

Pectate lyase is a hydrolytic enzyme used by diverse industries to clarify food. The enzyme occupies a 25% share of the total enzyme used in food industries, and their demand is increasing gradually. Most of the enzymes in the market belong to the fungal origin and take more time to produce with high viscosity in the fermentation medium, limiting its use. The bacteria belonging to the genus *Bacillus* have vast potential to produce diverse metabolites of industrial importance. The present experiment aimed to isolate pectate lyase-producing bacteria that can tolerate an alkaline environment at moderate temperatures. *Bacillus subtilis* PKC2, *Bacillus licheniformis* PKC4, *Paenibacillus lactis* PKC5, and *Bacillus sonorensis* ADCN produced pectate lyase. The *Paenibacillus lactis* PKC5 gave the highest protein at 48 h of incubation that was partially purified using 80% acetone and ammonium sulphate. Purification with 80% acetone resulted in a good enzyme yield with higher activity. SDS-PAGE revealed the presence of 44 kDa molecular weight of purified enzyme. The purified enzyme exhibits stability at diverse temperature and pH ranges, the maximum at 50 °C and 8.0 pH. The metal ions such as Mg^2+^, Zn^2+^, Fe^2+^, and Co^2+^ significantly positively affect enzyme activity, while increasing the metal ion concentration to 5 mM showed detrimental effects on the enzyme activity. The organic solvents such as methanol and chloroform at 25% final concentration improved the enzyme activity. On the other hand, detergent showed inhibitory effects at 0.05% and 1% concentration. Pectate lyase from *Paenibacillus lactis* PKC5 had *K*m and *V*max values as 8.90 mg/ml and 4.578 μmol/ml/min. The Plackett–Burman and CCD designs were used to identify the significant process parameters, and optimum concentrations were found to be pectin (5 gm%) and ammonium sulphate (0.3 gm%). During incubation with pectate lyase, the clarity percentage of the grape juice, apple juice, and orange juice was 60.37%, 59.36%, and 49.91%, respectively.

## Introduction

Pectin and pertinacious materials are the matrices of D-galacturonic acid joined by α-(1–4)-linkage and major components of the middle lamella of plant tissues^1^. It comprises various carbohydrates viz, arabinose, rhamnose, xylose, and galactose joined to the acidic backbone and accounts for almost 4% weight of the fresh fruits and major constituents of fruits, fibers, and vegetables^2^. The apple, carrot, and citrus fruits have the highest percentages of pectin, ranging from 32.0 to 35.0%^3^. Their peel and pulp contain significant portions of pectin, which provide strength and stability to the tissue^4^. The pectin parts must be removed from the pulp and peeled for industrial application.

Pectate lyase (PL) (E.C. 4.2.2.2.) is an enzyme that catalyzes the β-elimination of de-esterified pectin made up of acidic α-1–4 linked polygalactosyluronic; this results in the formation of unsaturated 4,5-d-galacturonate as the end product via transelimination^5^. The enzyme belongs to the PL1 family that does not require galacturonic acid residues' methyl esterification. Pectate lyase of PL9 and PL1 family act exogenously and endogenously, respectively^6^. Pectate lyase of the PL1 family produced by bacteria is the most studied endo-acting enzyme. . Polygalacturonase is the most abundant pectinolytic enzyme which hydrolyses α-1, 4-glycosidic bonds in pectic substances^7^.

Pectin degrading enzymes like Polygalacturonase, pectate lyase, and pectin methyl esterases are produced predominantly by *Bacillus* sp. And have been reported by various researchers^8–11^. However*,* many researchers have not explored Paenibacillus lactis for its pectate degrading ability, which could be a suitable candidate for juice clarification.

Enzymes are the main components for the textile, pulp, pharmaceutical, paper, and food industries. The food and beverage processing enzyme market size was around 1417.7 million USD in 2020 and would likely reach 1657.3 million USD by 2027. Of these food enzymes, pectinase or pectate lyase accounts for 25% market share, and these enzymes are produced by microbial fermentation. Pectate lyase enzymes are used in different applications like extraction and clarification of the fruit juices^12,13^, degumming of fibers^14^, bleaching of paper pulp^15^, coffee and tea fermentation^16^, and oil extraction^17^. Alkaline pectate lyase is usually produced by bacteria but made by some filamentous fungi and yeasts^18^. Marketable enzymes producing bacterial strains are always favored over fungal strains due to ease of fermentation procedure, purification process, scale-up, knowledge of strain handling and improvements, and cheap source of nutrients^19^.

Pectin is broken down by applying the enzymes to the fruit pulp; thus, the viscosity decreases due to softening and solubilization of the fruit pulps and increasing clarification. The traditional methods of clarifying pectin involve various steps such as centrifugation separation, which sometimes leads to a change in the taste and aroma of the juice. Moreover, it involves a higher cost than the enzymatic process. The industrial application always demands a cost-efficient method; thus, enzyme manufacturer wants the product optimization condition to be standardized with low input cost and better purity. The pectate lyase enzymes with low production costs is still a new experiment area. An essential aspect during cost reduction may be succeeded by using high yielding strains, optimal fermentation environments, and inexpensive raw materials as a carbon and nitrogen source for growing microorganisms. The conventional method relies on optimizing culture media by altering a single component that seems laborious, leads to an error during analysis, and cannot infer the interaction effects of components. Therefore, Response surface methodology (RSM) plays an essential role in determining the interaction effects of each component with a limited number of flasks. This gives accurate results with concurrent analysis of every component. The present experiment also extended the finding by performing RSM to determine the cheapest source of raw materials for the highest pectate lyase activity under assay conditions.

## Material and methods

### Sample collection and screening of microorganisms

The soil samples were collected from the high saline area around Dandi seashore, Navsari, India. The samples were serially diluted to 10^4^ to 10^6^ and spread on the Luria–Bertani (LB) agar plates (pH 9.0) followed by incubating at 37 °C for 48 h. Different colonies were selected based on their colony morphology and subculture on LB media plates. The distinct and well separated organisms were streak on to primary screening plates (PSP) containing NaNO_3_ 0.5%, KCl 0.05%, MgSO4 0.05%, K_2_HPO_4_ 0.1%, tryptone 0.05%, pectin 1%, and Agar 1.5% (pH 9.0) and incubated at 37 °C for 48 h. One of the incubated plates from each organism was flooded with iodine-potassium iodine solution. The clear zone around the colonies indicated the pectate lyase producers. The colonies were re-streaked and again screened to further confirm the pectate lyase activity. The overnight grown culture with pectate lyase activity was maintained as glycerol stock (50%) and stored at −20 °C for further use.

### Molecular characterization of selected bacteria

The potential isolates were further studied for Phylogenetic analysis using 16S rRNA sequencing. Briefly, the genomic DNA was isolated by suspending organisms in Lysozyme-SDS-Proteinase K (LSP) buffer and incubated at two successive temperatures of 37 °C and 65 °C for 30 min. each followed by purification. The samples were observed on 1% agarose gel. The 10 ng of genomic DNA was amplified with 1 Picomole 27F primer (5’ AGAGTTTGATCCTGGCTCAG3’), 1 Picomole 1492R primer (5’ TACCTTGTTACGACTT 3’), 2 μl of assay buffer (10x), 1 unit of Taq DNA polymerase, 250 μM dNTP mix. The amplified product was resolved on 1.5% agarose gel and purified using a DNA purification kit (SLS Research Ltd. Surat, India). The fragmentswere sequenced at SLS Research Ltd. Surat, India, and submitted to the NCBI using Banklt to assign accession numbers (https://www.ncbi.nlm.nih.gov). The neighbor-joining method developed a phylogeny tree using the Molecular Evolutionary Genetics Analysis software (MEGA 7) tool^20^.

### Estimation of pectate lyase activity

The pectate lyase activity was measured by incubating 100 μl of crude Protein with 900 μl of assay buffer containing pectin 1% at 50 °C for 10 min. The amount of galacturonic acid released under assay conditions was estimated following the Dinitrosalicylic acid (DNSA) method^21^. The absorbance of the reaction was measured at 540 nm on a spectrophotometer (Model UV-2600; Shimadzu, Japan). The pectate lyase activity was determined by comparing the values with the standard graph prepared from the known concentration of galacturonic acid. The total protein was measured using the Lowry method^22^ by considering absorbance at 650 nm. The standard graph was prepared using bovine serum albumin as standard. One unit of pectate lyase activity was monitored by the amount of enzyme required to liberate 1 μmol of galacturonic acid per ml per minute under assay conditions (1U = 1 μmol/ml/min). Specific activity was defined by the enzyme unit per milligram (mg) of Protein.

### Preparation of inoculum and production media

The overnight grown culture was monitored for their absorbance. The suitably diluted culture with OD 0.1 at 600 nm was transferred to a 150 ml flask containing 50 ml PSP production media. The inoculated flasks were incubated at 180 rpm at 37 °C. After incubation of 48 h, production media was centrifuged at 8000 rpm for 20 min at 4 °C for removal of microbial cells and debris. The supernatant was collected and used as a crude enzyme source for further characterization.

### Protein extraction and partial purification of the enzyme

The crude protein extracted from the procedure, as mentioned earlier, was further purified using two different methods. The crude protein was mixed with three volumes of ice-cold 80% acetone and 60% (w/v) Ammonium sulphate separately. Then, the mixture was kept at 4 °C overnight, followed by centrifugation at 8000 rpm for 15 min. The precipitate of crude proteins was suspended with 0.1 M Tris buffer (pH 8.5). The protein samples were dialyzed three times at lower temperatures by repeatedly changing the buffer. This step provides partially purified enzymes.

### Biochemical and functional characterization of pectate lyase determination of optimum pH, temperature, and substrate concentration

The reaction mixture containing partial purified enzyme and substrate was incubated at different temperatures (30 °C, 37 °C, 50 °C, 60 °C, and 70 °C) for 20 min. Similarly, the substrate was dissolved in various buffer (0.1 M sodium phosphate buffer for pH 6.0 and 7.0, 0.1 M Tris buffer for pH 8.0, 9.0, and 10.0) and incubated with purified enzyme for 20 min. The reaction product was evaluated by the DNS method. Enzyme was incubated with different concentration of substrate (0.1%, 0.5%, 1.0%, 1.5%, 2.0%, 2.5%, and 3.0%) to determine *K*_*m*_ and *V*_max_ of the enzyme using Michaelis–Menten equation. The plot of 1/V versus 1/S was used to get optimum substrate concentration for better enzyme activity.

### Effect of metal ions, inhibitors, and organic solvents on enzyme activity

To study the effect of various activators and inhibitors on the activity of the enzyme, the pectate lyase was incubated for 1 h with 1 mM and 5 mM concentrations of different metal ions such as Ca^2+^, Cu^2+^, Co^2+^, Mg^2+^, Mn^2+^, Zn^2+^, Fe^2+^, Hg^2+^, Na^2+^ and Ba^2+^. Following the incubation, the enzyme activity was measured under standard assay conditions. The organic solvents such as acetone, ethanol, DMSO, isopropanol, chloroform, and methanol at 25% final concentrationwere mixed with partially purified enzyme for 4 h; effects were evaluated as a change in relative enzyme activity. The relative activity (%) was determined by dividing the enzyme activity in the presence of mentioned compounds to the enzyme activity in control. Similarly, various detergents including SDS w/v (1%), tween 20, EDTA, and β mercaptoethanol in v/v (1%) were incubated with partially purified enzymes for 1 h followed by their activity evaluation under standard assay condition.

### Molecular weight analysis using SDS PAGE

The molecular weight of the partially purified enzyme was determined by performing 12% sodium dodecyl sulfate–Polyacrylamide gel electrophoresis (SDS-PAGE) at 25 mA in the presence of Tris–Glycine buffer. The isolated protein bands were stained using CBB dye (0.25% Coomassie Brilliant Blue R-250, 50% methanol, and 10% glacial acetic acid) for 3–4 h, followed by the de-staining solution containing 50% methanol and 10% glacial acetic acid. The molecular weight was compared with known standard loaded in the first lane.

### Statistical optimization of pectate lyase production

#### Assessing the significant media components for pectate lyase production using Plackett–Burman [PB] methodology

The significant media components were screened for the Pectate lyase production by the multifactorial PB design^23^. The variables included in the study are pectin, NaNO_3_, (NH_4_)_2_SO_4_, tryptone, MgSO_4_, KH_2_PO_4_, K_2_HPO_4_, CaCl_2_, and FeSO_4_, and their respective higher (+) and lower (−) concentrations for PBD are presented in Table 1.

**Table 1:** Media components and their levels for the Plackett–Burman experiments.

Variables	Components (gm%)	+	-
X1	Pectin	1.0	0.25
X2	NaNO_3_	2.0	0.25
X3	(NH_4_)_2_SO_4_	5.0	1.0
X4	Tryptone	2.0	0.50
X5	MgSO_4_	0.3	0.05
X6	KH_2_PO_4_	1.0	0.05
X7	K_2_HPO_4_	1.0	0.05
X8	CaCl_2_	0.25	0.01
X9	FeSO_4_	0.5	0.1

Total 12 runs design having nine media components and two dummies for their higher and lower concentration considered for the experiment were depicted in Table 5, 6. Entire experiments were carried out in a 25 ml media system in a 250 ml Erlenmeyer flask.

The 0.1 OD of the culture was inoculated, followed by incubation for 48 h at 37 °C. After incubation, the enzyme was harvested, and pectate lyase production was evaluated using standard assay and represented as μmol/ml/min. The Plackett–Burman design is of first-order reaction, and the effect of each variable on the pectate lyase production was determined. The standard error (SE) and the significances (*p*-value) of each variable concerning their concentration were also calculated to identify the essential media components that drive the Pectate lyase production. The variables with confidence levels greater than 99% were only considered to influence the enzyme production significantly.

#### Level optimization of the screened components using response surface central composite design (RSM-CCD)

The central composite design was adopted to observe the interactive effects of the media components, which were found significant in the Plackett- Burman design (Table 5, 6). The minimum and maximum values of the significant variable for the RSM-CCD experiment were decided from the Placket-Burman experiment. The five different pectin and ammonium sulphate levels were considered to enhance pectate lyase production using *Paenibacillus lactis* PKC5 through the CCD experiment and are depicted in Table 2.

**Table 2:** Level of the significant media component chosen for the RSM-CCD experiment.

Variables	Level of the components adopted for CCD				
	-α	-1	0	+ 1	+ α
Pectin	-0.43	0.5	2.75	5	5.93
Ammonium sulphate	-0.25	0.3	1.65	3	3.55

CCD is a factorial design where the optimal response of the system is depicted in the form of counterplots depending on linear or quadratic effects of the key components and is also supported by the model equation. CCD comprises two F factorial points, 2 axial points (±*α*), and natural center points. The entire experiments were outlined using Design Expert Software, v10 (Stat- Ease Inc; USA). A total of 14 experimental runs (Table 8) were designed having a varying level of two media components, i.e., pectin and ammonium sulphate, keeping the rest of the experimental condition constant (Table 9). The experiment was conducted in a 50 ml media system in the 250 ml of Erlenmeyer flasks followed by incubation for 48 h at 37 °C. The enzyme production was estimated using the standard assay procedure as described earlier.

#### Application of pectate lyase for clarification of various juices (% clarity)

Fresh apple, orange, and grapefruit juices were collected from the market and incubated at 65 ℃ for 45 min to inactivate the enzymes and then stored at 4 ℃. The juices were treated with partially purified pectate lyase (1U/ml) and incubated at 50 ℃ for 1 h. enzyme additionThe juice without adding enzyme was considered as a control and assayed under the same condition as the sample. Following the incubation, the juices (test and control) were incubated at 85 ℃ for 5 min to stop the reactions. The juice clarity was determined by tacking absorbance at 440, 520, and 660 nm for apple juice, 420, 520, and 660 nm for grape juice, and 660 and 540 nm for the orange juice^24^.

$$\mathrm{Clarity }\left(\mathrm{\%}\right)=\frac{\mathrm{OD of untreated juice}-\mathrm{OD of treated Juice}}{\mathrm{OD of untreated juice}}\times 100$$

where, OD of Untreated Juice is the sample with no enzyme and incubation at 50 °C, OD of Treated Juice is a sample with enzyme Incubated at 50 °C.

## Results

### Screening of bacteria

**Figure 1:**
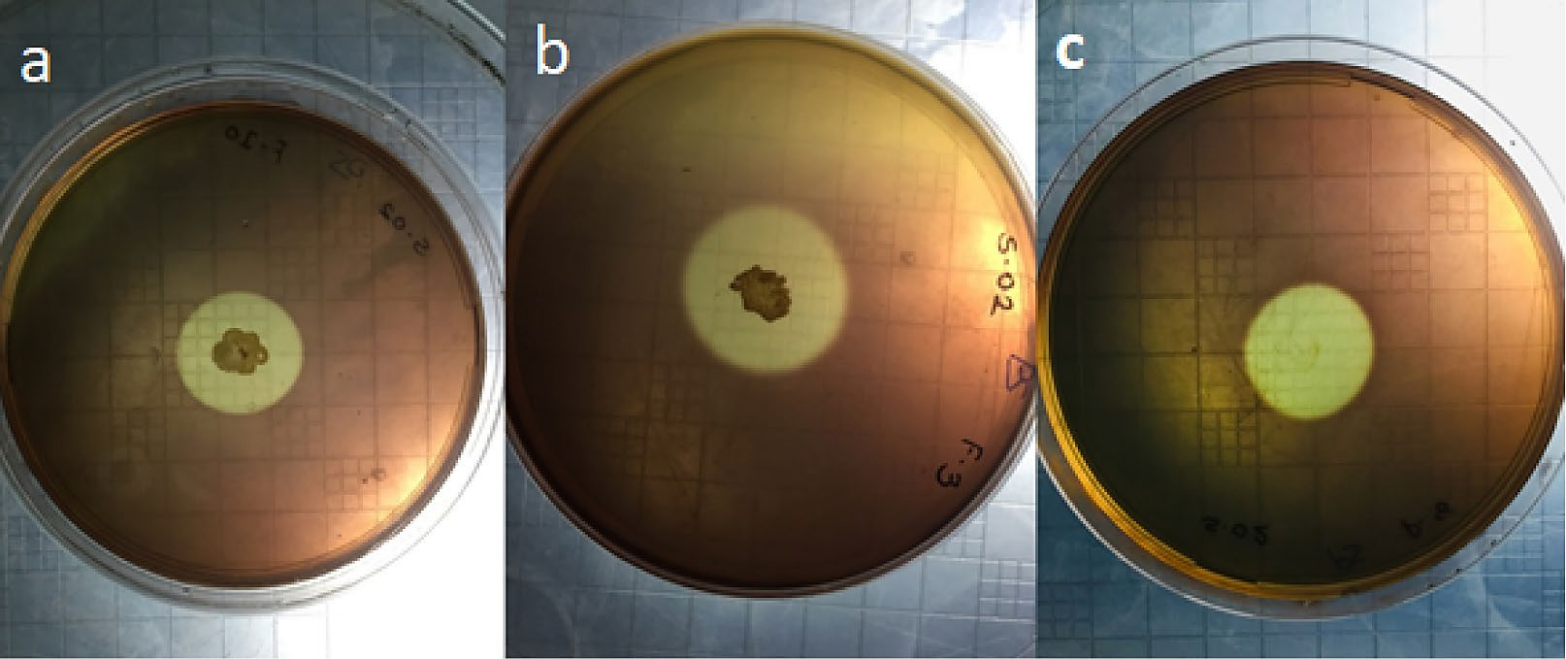
Zone of Pectin hydrolysis on PSP agar medium of isolate (**a**) *Bacillus licheniformis* PKC4, Centre (**b**) *Bacillus subtilis* PKC2, (**c**) *Paenibacillus lactis* PKC5 after Gram’s iodine staining.

Most of the previous reports showed pectate lyase belongs to fungi origin, and less number of papers deals with the alkaline Bacteria as a source of enzyme. The present study aimed to isolate and screen alkaline bacteria for their potential to cleave pectate under alkaline conditions and media optimization using RSM and characterization of partially purified enzymes. The present experiment was initiated by collecting rhizospheric soil from the plants viz. *Citrus aurantifolia, Syzygium cumini,* and the hybrid Kinnow mandarin. There were initially 50 distinguished colonies observed on the plates. Out of these isolates, only four isolates were found to be most pectinolytic by iodine assay and observed as a clear zone around the colony (Fig. 1). The colony diameter, as well as the hydrolytic zone, was calculated after 48 h of incubation. The two organisms named PK2 and PK5 gave similar and highest zone of lysis (Table 3).

**Table 3:** Colony diameter and zone of hydrolysis.

Bacterial strain	Colony diameter (mm)	Zone pf pectin hydrolysis (mm)
*Bacillus subtilis* PKC2	5.0	10.0
*Bacillus licheniformis* PKC4	4.0	7.0
*Paenibacillus lactis* PKC5	3.0	10.0

### Molecular analysis of selected microbes

The four organisms, PK2, PK4, PK5, and ADCN, were further characterized at the molecular level using 16S rRNA gene sequences obtained from SLS research Pvt. Ltd. The sequences were aligned to the NCBI database using the BLAST tool and showed above 90% homology with *Bacillus* spec. In the database. The nucleotide sequences of the respective organism were submitted to the GeneBank using the Banklt tool to obtain accession numbers. The Phylogenetic tree (Supplementary Figure S1a, S1b, S1c, and S1d) was constructed using the neighbor-joining method through the MEGA 7.0 tool by considering 16 closely related sequences that appeared during local alignment from GeneBank Database.

The bootstrap consensus tree obtained from 1000 replicates and evolutionary distances were analyzed using the maximum composite Likelihood method. The numbers mentioned at the nodes are considered bootstrap support values of the tree obtained by 1000 inferred replications. The gaps and missing values were ignored during the analysis. The *Bacillus subtilis* PKC2, *Bacillus licheniformis* PKC4, and *Bacillus Sonorensis* ADCN formed distinct clades among the sequences taken for the analysis and thus could be considered as new strains. The results indicated that *Paenibacillus lactis* PKC5 showed 100% similarity with *Paenibacillus lactis* strain MB1871 in Phylogenetic analysis.

### Production of pectate lyase and activity determination

**Figure 2:**
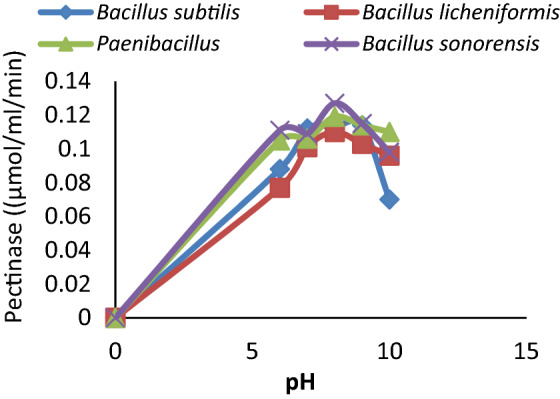
Effect of various pH on the activity of pectinase.

The four organisms were further evaluated based on pectate lyase activity at diverse Temperatures and pH. The *Bacillus subtilis* PKC2 showed the highest activity at pH 9.0 and kept its 98.25% and 99.12% activity at pH 7.0 and pH 8.0, respectively while, 61.04% activity was observed at pH 10.0. *Bacillus licheniformis* PKC4, *Paenibacillus lactis* PKC5, and *Bacillus Sonorensis* ADCN gave the highest enzyme activity at pH 8.0 (Fig. 2). Further increase in pH had a negative impact on its activity.

**Figure 3:**
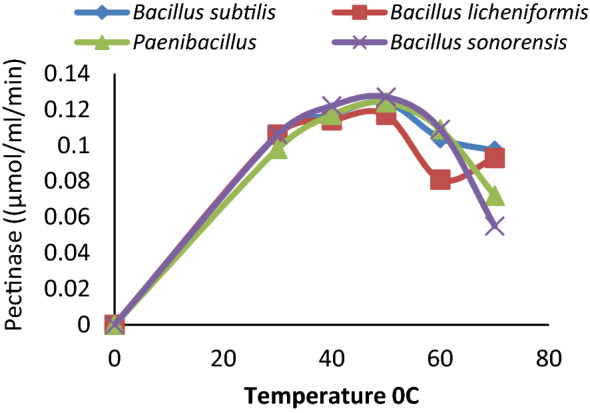
Effect of temperature on the activity of pectinase.

The Pectate lyase activity from *Paenibacillus lactis* PKC5 was observed to be 88.24%, 89.00%, 95.80%, and 92.44% at pH 6.0, 7.0, 9.0, and 10.0 respectively. The stability of enzymes at different pH was considered advantageous for their industrial application; hence *Paenibacillus lactis* PKC5 was selected for further characterization and applications. Pectate lyase activities from all the isolates were examined for their optimum temperature of pectin hydrolysis. The enzyme showed activity between 30 and 70 °C and was found to be maximum at 50 °C. There was a slight decrease in the enzyme activity of *Paenibacillus lactis* PKC5 with changes in the temperature and keeps 79.03% activity at 30 °C and 58.06% activity at 70 °C (Fig. 3). A similar pattern of activity at diverse conditions was observed from the different organisms under study. Therefore, the enzyme Pectate lyase from *Paenibacillus lactis* PKC5 was moderately thermotolerant and alkaline.

### Enzyme purification

The enzyme production was carried out in the PSP media with 1% pectin as a sole carbon source in an orbital shaker incubator for 48 h. The media from incubated flask were centrifuged at 8000 rpm for 15 min to obtain cell-free supernatant. The supernatant was considered a crude enzyme and partially purified using two separate methods. The purification summary of pectate lyase is mentioned in Table 4. It was inferred from the table that the acetone method gave 7.36 mg protein while ammonium sulphate based purification yielded 10.6 mg protein. The dialyzed sample also showed the same trend of purification. There was not much difference in the total activity, but specific activity was higher with acetone (29.97 U/mg) based purification step compared to ammonium sulphate method (15.80 U/mg). This table and the purification summary values indicated that the acetone-based method had decent advantages over the ammonium sulphate method for active enzyme purification. The partially purified enzyme was further evaluated for its biochemical and functional characterization.

**Table 4:** The partial purification summary of alkaline Pectate lyase.

Purification step	Total Vol. (mL)	Total protein (mg)	Total activity (U)	Specific activity (U/mg)	Yield (%)	Purity fold
Crude broth	50	33	63.5	1.92	100	1
Acetone	20	7.36	23.19	3.15	36.52	1.64
(NH4)_2_SO_4_	20	10.6	24.40	2.30	38.43	1.20
Dialyzed acetone	10	0.72	21.58	29.97	33.98	15.61
Dialyzed (NH_4_)_2_SO_4_	10	1.2	18.96	15.80	29.85	8.23

### Biochemical and functional characterization of pectate lyase

#### Effects of substrate concentrations, michaelis–menten constant (Km) and Vmax

**Figure 4:**
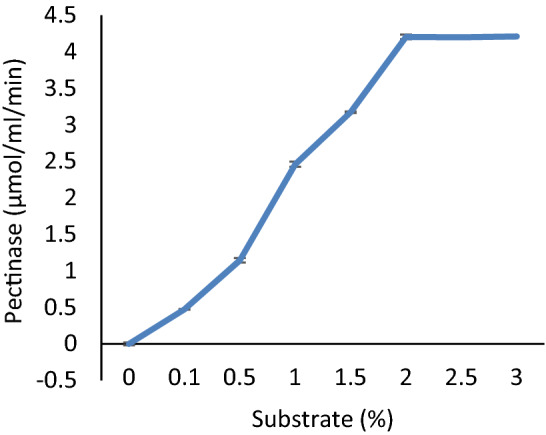
Effect of substrate-level on pectinase activity.

**Figure 5:**
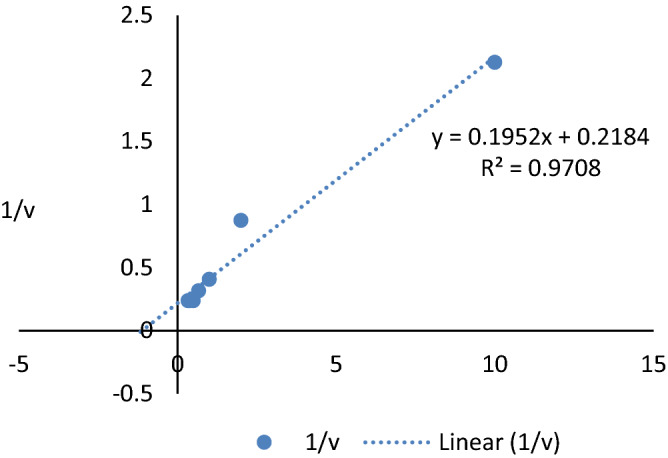
Michaelis–Menten kinetics.

The enzyme activity was increased as soon as the substrate concentration increased to 2–3% pectin. Further increases in the pectin exhibited little hike in the activity, and thus enzymes active site was occupied (Fig. 4). The enzyme was incubated with different substrate concentrations, and reaction products were analyzed by released of galacturonic acid. The regression coefficient R^2^ was 0.970, indicating positive correlations between experimental parameters. The non-regression analysis of the reaction gave the enzyme kinetic values viz. *K*m as 8.90 mg/ml and *V*max as 4.578 μmol/ml/min (Fig. 5).

#### Effect of metal ions, inhibitors, and organic solvents on pectate lyase activity

**Figure 6:**
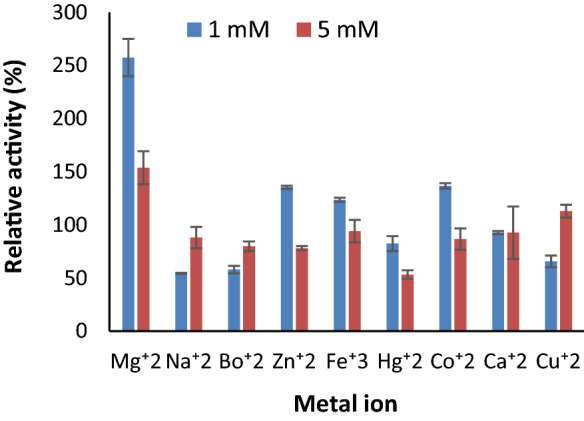
Effect of various metal ions at 1 mM and 5 mM concentrations on pectate lyase activity of *Paenibacillus lactis* PKC5.

**Figure 7:**
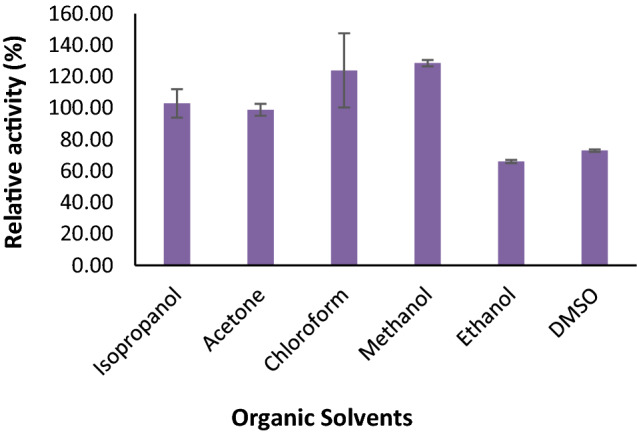
Effect of different organic solvents on pectate Lyase activity from *Paenibacillus lactis* PKC5.

**Figure 8:**
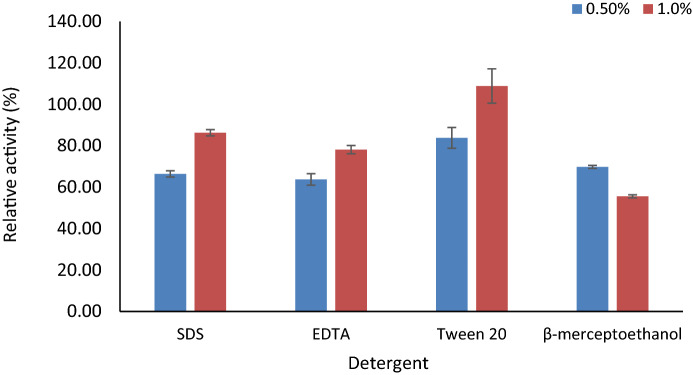
Effect of different detergents on pectate lyase activity from *Paenibacillus lactis* PKC5.

There was a significant increase in the pectate lyase activity upon incubation with Co^2+^, Mg^2+^, Zn^2+^, Fe^2+^; in particular, Mg^2+^ ions increased activity by 268.46%, while Ca^2+^, Cu^2+^, Mn^2+^, Hg^2+^, Na^2+^, and Ba^2+^ reduced its activity at 1 mM concentration. Increasing metal ion concentrations to 5 mM reduced the enzyme activity by 20% to 30% from their 1 mM counterpart (Fig. 6). Thus, 1 mM concentration of some metal ions could be a cofactor for the enzyme-catalyzed reaction. Without adding any components like metal ions, organic solvents, and detergents, the enzyme activity was considered 100%. The enzyme activity was enhanced by 129.97% in methanol (25%) and 105.82% in chloroform (25%), while the other organic solvents viz. Acetone, Ethanol, DMSO (Dimethyl sulphoxide), isopropanol decreased enzyme activity during 4 h of incubation. In the presence of isopropanol and acetone, the enzyme has retained 96% of its activity (Fig. 7). The enzyme activity was also affected by inhibitors at various concentrations. The 1% concentration of inhibitors such as SDS, EDTA, and β mercaptoethanol reduces enzyme activity; in particular, β mercaptoethanol decreased activity by 55% while Tween 20 showed an increase in the activity by 114.00% (Fig. 8). The 0.5% of mentioned inhibitors have a more predominant impact on enzyme activity as the higher concentration could have a role in simplifying Protein's three-dimensional structure for better activity. Apart from β Mercaptoethanol, all the inhibitors showed 20–35% lower activity at 0.5% concentration.

### SDS-PAGE analysis

**Figure 9:**
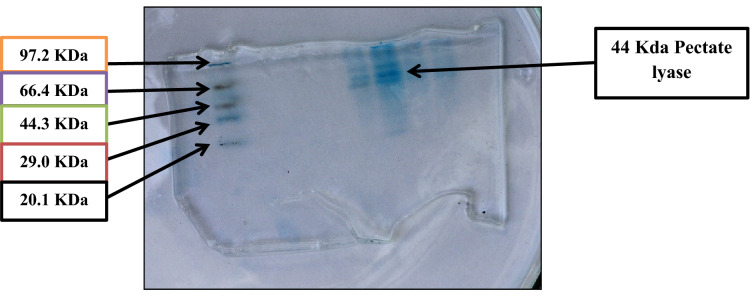
SDS–polyacrylamide gel electrophoresis of pectate lyase from *Paenibacillus lactis* PKC5.

SDS-PAGE of the partially purified samples was carried out to determine the purity and molecular weight of the Pectate lyase withhe molecular protein marker in the range from14.2 to 94.7 kDa. SDS-PAGE analysis of pectate lyase showed a molecular mass of approximately 44 kDa (Fig. 9) for pectate lyase.

### Evaluating the significant media components through PBD and their level optimization using RSM-CCD

The screenings of the significant variables which induce the pectate lyase production were evaluated using ANOVA of the Plackett–Burman experiment. The entire experimental run comprises 12 sets where X1–X9 were variables, and X10–X11 were dummy. Here, the positive and negative sign of Exi (concentration effect of tested variables) shows the influence of the variable on pectate lyase yields. Positive (Exi value) indicates the influence of a particular component is more significant at high concentration, while a negative (Exi value) means the influence of variable is more significant at low concentration. In the present investigation two-variable, i.e., pectin (X1) and ammonium sulphate (X3), are selected for further level optimization strategy using RSM-CCD as they showed > 95% of confidence. In comparison, the rest of 7 components have not shown > 95% of confidence in ANOVA. The effect (Exi), txi, *p*-value, and (%) confidence level of each component is shown in Tables 5, 6.

**Table 5:** Plackett–Burman design matrix where X1, X2…..X9 indicate a number of variables chosen for the study and D1, D2 are dummies for the 12 runs multifactorial design.

Run No	Pectin	NaNO_3_	(NH_4_)_2_SO_4_	Tryptone	MgSO_4_	KH_2_PO_4_	K_2_HPO_4_	CaCl_2_	FeSO_4_	Dummy		Pectate lyase (μMl/ml/min )
	X1	X2	X3	X4	X5	X6	X7	X8	X9	D1	D2	
1	1.25	2.00	1	2	0.3	0.05	1	0.01	0.1	+	−	0.2210
2	0.25	2.00	5	0.5	0.3	1	1	0.01	0.1	−	+	0.0200
3	1.25	0.25	5	2	0.05	1	1	0.25	0.1	−	−	0.0754
4	0.25	2.00	1	2	0.3	1	0.05	0.25	0.5	−	−	0.0465
5	0.25	0.25	5	0.5	0.3	0.05	1	0.25	0.5	+	−	0.0100
6	0.25	0.25	1	2	0.05	1	1	0.01	0.5	+	+	0.0398
7	1.25	0.25	1	0.5	0.3	1	0.05	0.25	0.1	+	+	0.1432
8	1.25	2.00	1	0.5	0.05	0.05	1	0.25	0.5	−	+	0.1850
9	1.25	2.00	5	0.5	0.05	1	0.05	0.01	0.5	+	−	0.1309
10	0.25	2.00	5	2	0.05	0.05	0.05	0.25	0.1	+	+	0.0333
11	1.25	0.25	5	2	0.3	0.05	0.05	0.01	0.5	−	+	0.1629
12	0.25	0.25	1	0.5	0.05	0.05	0.05	0.01	0.1	−	−	0.0376

**Table 6:** Analysis of variance for Plackett–Burman design (PBD).

No	Variables	Effect	SE	t value	*p*-value	% significance
X1	Pectin	0.121853	0.0095	13.54	0.005	99.4589
X2	NaNO_3_	0.028001	0.0095	3.11	0.090	91.03636
X3	(NH_4_)_2_SO_4_	-0.04009	0.0095	-4.45	0.047	95.31181
X4	Tryptone	0.008703	0.0095	0.97	0.436	56.44336
X5	MgSO_4_	0.016927	0.0095	1.88	0.201	79.92596
X6	KH_2_PO_4_	-0.00054	0.0095	-0.06	0.958	4.209175
X7	K_2_HPO_4_	-0.03234	0.0095	-3.59	0.069	93.05148
X8	CaCl_2_	-0.01978	0.0095	-2.20	0.159	84.0923
X9	FeSO_4_	0.007425	0.0095	0.83	0.496	50.39125

Plackett–Burman's design screened the significant media components, but the combined effect of those variables was determined using the response surface central composite design (RSM-CCD). The experiment was conducted in 14 runs set up under optimized conditions as described in Table 7, and the pectate lyase production response was recorded. The experimental response was evaluated using multiple regression analysis through a quadratic model, and the polynomial equation was used for obtaining the maximum pectate lyase production –

$$ Y = 0.237841 + 0.376193 \times A + 0.011604 \times B - 0.0912 \times AB + 0.158294 \times A^{2} + 0.04875 \times B^{2} $$

where Y is the response factor (Pectate lyase production; μmol/ml/min), A is pectin (gm%); B is ammonium sulphate (gm%).

**Table 7:** Design matrix of 14 runs generated through RSM-CCD (Design-Expert software V10).

Run No	Factor 1	Factor 2	Response 1
	A: pectin (gm%)	B: ammonium sulphate (gm%)	Pectinase activity (μmol/ml/min)
1	0.50	0.30	0.0282
2	5.00	0.30	1.0090
3	0.50	3.00	0.1730
4	5.00	3.00	0.7890
5	-0.43	1.65	00
6	5.93	1.65	0.9990
7	2.75	-0.25	0.2210
8	2.75	3.55	0.3398
9	2.75	1.65	0.1990
10	2.75	1.65	0.2793
11	2.75	1.65	0.2677
12	2.75	1.65	0.2010
13	2.75	1.65	0.3010
14	2.75	1.65	0.1790

The results obtained from the analysis of variance (ANOVA) for investigation of the obtained model accuracy are presented in Table 8.

**Table 8:** ANOVA for response surface quadratic model for the pectate lyase production.

Source	Sum of squares	df	Mean square	F-value	*p*-value Prob > F	
Model	1.361486	5	0.272297	44.73552	1.23E-05	Significant
A-pectin	1.132169	1	1.132169	186.0033	8.04E-07	Significant
B-ammonium sulphate	0.001077	1	0.001077	0.176967	0.685066	
AB	0.033267	1	0.033267	5.465455	0.047576	Significant
A^2^	0.185036	1	0.185036	30.39942	0.000565	Significant
B^2^	0.01755	1	0.01755	2.883277	0.127935	
Residual	0.048695	8	0.006087			
Lack of fit	0.035764	3	0.011921	4.609583	0.0666	Not significant
Pure error	0.012931	5	0.002586			
Cor total	1.410181	13				

In the quadratic model, the calculated amount of experimental F is way more than its critical amount in the level of significance (95%), which suggests the model’s significance. Also, the value of *P* is related to the models, which are less than 0.05 (on the significance level of 95%) that is indicative of the model's efficiency for the prediction of experimental results. The Model F-value of 44.74 implies that the model is significant. Values of "Prob > F" less than 0.0500 indicate model terms are significant. In this case, A, AB, A^2^- are significant model terms. The "Lack of Fit F-value" of 4.61 implies there is a 6.66% chance that a "Lack of Fit F-value" this large could occur due to noise, and here in our data set, Lack of fit is found to be non-significant, which is indirectly indicating that the obtain data fit to the model is very well ordered. The multiple correlation coefficient (R^2^) value near 1 indicates a better correlation between the predicted and observed values. The "Pred R-Squared" of 0.8065 is in reasonable agreement with the "Adj R-Squared" of 0.9439, while the R-Squared was found to be 0.97. "Adeq Precision" measures the signal-to-noise ratio. A ratio greater than 4 is desirable, and the presented experiment yielded a ratio of 21.939 indicates an adequate signal. This model can be used to navigate the design space.

**Figure 10:**
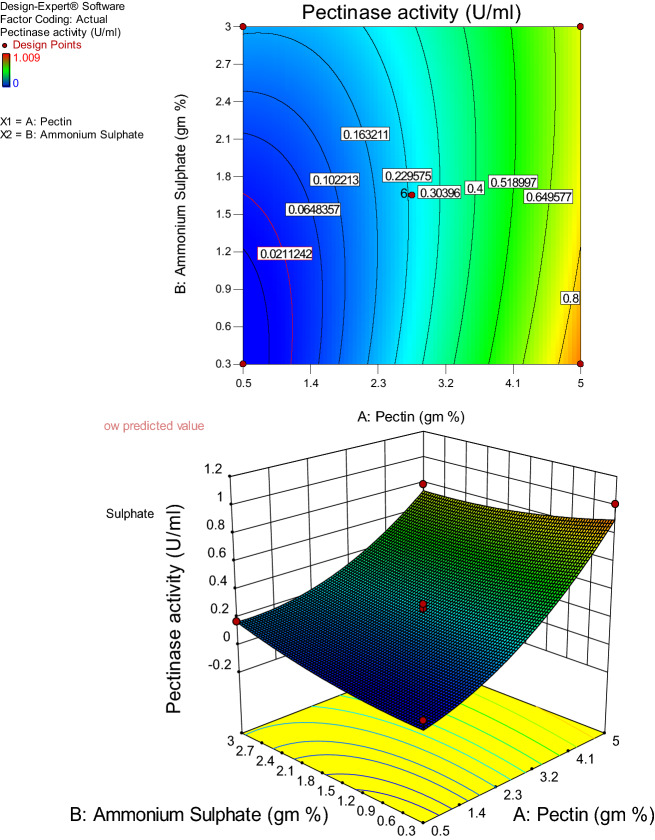
Contour plot (**A**) and response surface plot (**B**) of the interactive effect of ammonium sulphate and pectin on the Pectate lyase production by *Paenibacillus lactis* PKC5.

3D response surface (and contour) plots were examined, and their impact was estimated to check the interaction between diverse variables and their effect on enzyme production. The primary purpose of response surface plots is to delineate two factors at a time while maintaining other factors at fixed levels. The interactive effect of the selected parameters, i.e., pectin and ammonium sulphate, to maximize the pectate lyase production were evaluated and depicted in the response surface plot and contour plot (Fig. 10).

It was observed that both the components at their lower level did not show any notable effect to uplift the Pectate lyase production. It was also observed that keeping the pectin at its lower level and gradually increasing the ammonium sulfate concentration did not significantly affect the Pectate lyase production.

Moreover, ammonium sulphate at its higher selected level and pectin at its higher level with a concomitant increase in the ammonium sulphate can uplift the pectate lyase production to a certain extent. However, maximum enhancement in the Pectate lyase production was only observed when ammonium sulphate was kept at its lower level and pectin concentration at its higher level, as depicted in Fig. 10.

### Validation of the quadratic model

Validation of the level of pectin and ammonium sulphate predicted by the response surface model in the production medium was carried out. The optimum concentrations of pectin and ammonium sulphate were predicted to be 5 gm% and 0.3 gm%, respectively, through the model graph. The predicted yield of Pectate lyase obtained from the model using these optimum concentrations of the two components was 0.91 U/ml. During the validation experiments, the actual Pectate lyase production was 1.20 U/ml, which is higher than the predicted value. Therefore, this response surface model was reliable for predicting higher Pectate lyase production by *Paenibacillus lactis* PKC5.

### Effect of pectate lyase on clarification of fruits juices

**Figure 11:**
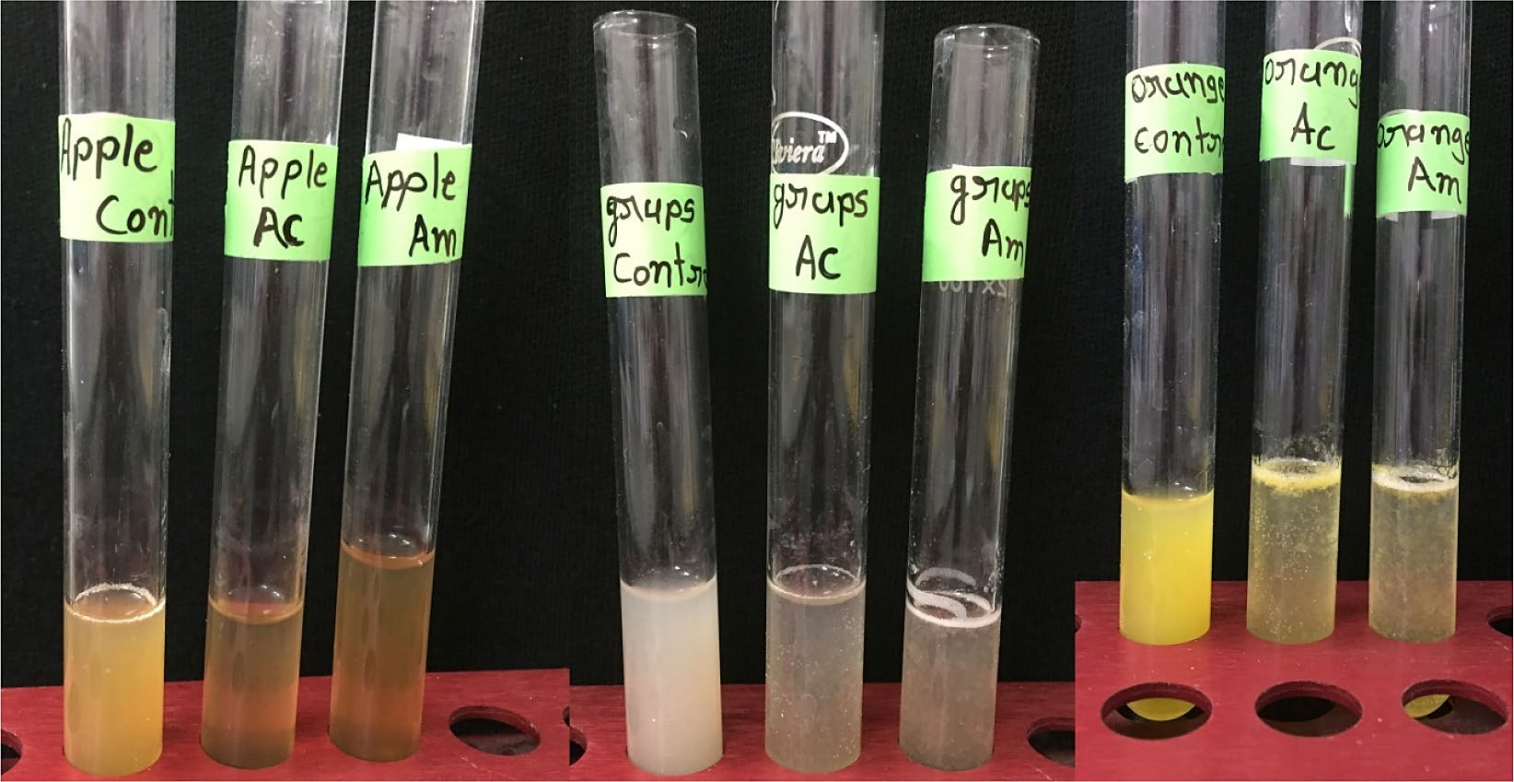
Application of Pectate lyase for fruit juice clarification Left, Centre, and Right.

The clarity percentage of the grape juice, apple juice, and orange juice was 60.37%, 59.36%, and 49.91%, respectively, under the mentioned assay condition (Table 9). The higher clarification percentage indicated that the enzyme could be helpful for juice preparation at the commercial level (Fig. 11).

**Table 9:** Effect of pectate lyase enzyme on clarification of juices.

Clarity (%)	Apple juice	Orange juice	Grape juice
Acetone purified	59.36	49.91	60.37
Ammonium sulphate purified	59.17	51.18	60.18

## Discussion

The present study aimed to determine the potent alkaline pectate lyase for their application in food industries. The soil samples were collected from the rhizospheres, and they contained diverse microbes with different enzymatic potential^25^. These soil microbes play an essential role in community management around the roots, maintaining soil fertility, and assisting in uptake nutrients^26^. The soil contains complex plants tissues that are hydrolyzed by the microbes by excreting pectinolytic enzymes. The alkaline pectinolytic enzymes have an immense industrial application such as in the paper and pulp industry for better printability, pectic waste^27^, degumming and scouring of textile materials^28^, tea fermentation, and removal of the coffee seed coat.

The Bacillus was the predominant genera found in the initial screening, and it was well-reviewed by Kavuthodi and Sebastian^29^. The *Paenibacillus lactis* PKC5 showed the highest hydrolysis zone in 1% pectin screening medium; hence pectate lyase from this organism was further characterized. Moreover, there were few previous reports indicated that *Paenibacillus* genus as a source of pectinase^30–33^.

The organisms showed optimum growth at 37 °C indicated their mesophilic nature. The *Paenibacillus lactis* PKC5 produced maximum enzyme activity after 48 h of incubation, which signifies their possible use in industries. A shorter production cycle gives a significant cost advantage, and more cycles could be run per unit time. Another reason could be the accumulation of waste material and the utilization of media ingredients for growth. The pectin was used as the sole carbon source. The Pectate lyase from the *Bacillus subtilis* PKC2, *Bacillus licheniformis* PKC4, *Paenibacillus lactis* PKC5, and *Bacillus Sonorensis* ADCN showed pH stability and remains active between pH 6.0 and pH 10.0. The Pectate lyase of *Paenibacillus lactis* PKC5 was selected for further analysis due to the reason that it can withstand a wide pH range from neutral to alkaline and maintain 88.00% (1.05 μmol/ml/min) to 92.00% (1.10 μmol/ml/min) activity respectively with optimum activity at pH 8.0 (1.19 μmol/ml/min). Therefore, all organisms were potential candidates for pectate lyase, but pH plays a significant role in enzyme activity for industrial application; thus, *Paenibacillus lactis* PKC5 was selected. On the other hand, *Bacillus subtilis* PKC2, *Bacillus licheniformis* PKC4, and *Bacillus Sonorensis* ADCN were found to show better stability between temperatures from 30 (90%) to 70 °C (78%) compared to *Paenibacillus lactis* PKC5. The pectate lyase from *Paenibacillus lactis* PKC5 showed 0.98 μmol/ml/min enzyme activity at 30 °C and 0.72 μmol/ml/min activity at 70 °C respectively, while optimum activity was observed at 50 °C (1.24 μmol/ml/min). Therefore, the enzyme Pectate lyase from *Paenibacillus lactis* PKC5 was considered thermotolerant and alkaline. Akhinyemi^31^ has also carried out similar experiments by considering *Bacillus megaterium, Bacillus bataviensis, and Paenibacillus sp*. and found that *Bacillus* species showed optimum activity at pH 8.0 and 60 °C. At the same time, *Paenibacillus* sp. exhibited its activity at pH 6.5 at 40 °C. However, the result obtained in the experiment was in accordance with the result of other studies with slight divergence in the temperature and pH optima, which indicated that *Paenibacillus* sp. might require a different reaction environment^30–33^. The previous finding in Bacillus species^11,34–38^ supported the present result of alkaline pectate lyase with moderate thermophilic nature.

The purified enzyme was used to incubate with different concentrations of the substrate to determine the saturation point of the enzyme by the substrate. For the industrial application, lower Km value and higher Vmax are always advantageous as Km reflects the enzyme's affinity towards its substrate. Pectate lyase from *Paenibacillus lactis* PKC5 had *K*m and *V*max values as 8.90 mg/ml and 4.578 μmol/ml/min, respectively*.* It was inferred from the present results that little enzyme is sufficient to hydrolyze a large quantity of substrates until it is all site has been occupied. It was concluded from the present results that little enzyme is sufficient to hydrolyze a large quantity of substrates until its all site has been occupied. Manal^30^ reported the K_m_ and V_max_ was 0.772 mg/ml and 7.936 μmol/ml/min respectively from *Paenibacillus lactis* NRC1*.* Ouattara^39^ reported that Pel-22 from *Bacillus pumilus* BS22 had Km of 0.45 mg/ml and Vmax of 1.41 μmol/ml/min.

Similarly, pectinase produced by *Bacillus subtilis* strain Btk 27 had *K*m and *V*max values of 1.88 mg/ml and *V*max 149.6 IU, respectively^35^. The partially purified Pectate lyase from *Paenibacillus lactis* PKC5 was subjected to SDS PAGE analysis and found to be 44,000 Da, similar to the molecular weight reported from *Paenibacillus Xylanolyticus 2-6L3*^32^* and reported by most of the Pectate lyase from bacterial origin.* Likewise, Pectinase from *Paenibacillus lactis* NRC1 was seen as 45.00 KDa band by SDS PAGE^30^.

In contrast, Akinyemi^31^ showed that the molecular weight of pectinase purified from *Paenibacillus* sp. is 29,512 Da which was less than reported by the present paper. Moreover, pectinase from *Bacillus cereus* and *B. licheniformis* showed a lower molecular weight of 38,304.27 Da^40^. Metal ions act as cofactors and require the action of catalyst, and thus considered as metalloenzyme. They also maintain the active conformation of the enzyme to carry out the enzyme-catalyzed reaction. The present study at one mM final concentration of Co^2+^, Mg^2+^, Zn^2+^, and Fe^2+^ has significantly improved enzyme activity several-fold. The Mg^2+^ ion even at 5 mM showed improved activity while Cu^2+^ at 5 mM concentration stimulates the pectate lyase activity. A similar study was conducted by Oumer and Abate^35^ using *Bacillus subtilis* strain Btk27 and reported that *Pectinase activity* was improved by adding metal ions viz. Mg^2+^, Zn^2+^, Co^2+^, and Fe^2+^. Moreover, Khatri^41^ reported that pectinase activity from the Aspergillus flavus was greatly affected by the addition of Ca^2+^ and fairly by Na^+^, Pb^+2^, and Zn^2+^. These results indicated that enzymes from different sources require various metal ions at particular concentrations to carry out the activity. Moreover, the evolution of the enzyme also determines the metal ion requirement at the active site as organisms are evolved as per the environment around them for better growth and tend to modify their pathways or enzymes.

On the other hand, Sunnotel and Nigam^42^ found that the Ca^2+^ metal has an influential role on pectinase from *Bacillus *sp.* KSMp576.* Metal ions like Na^+^ and K^+^ improved pectinase activity produced by *Paenibacillus* sp. While Zn^2+^ and Mn2^+^ reduced the activity of enzyme^31^ However, these results are contrary to the observation obtained in the present study. Anggraini^43^ concluded that 2 and 4 mM concentrations of Mg^2+^ stimulate the pectinase activity while higher concentrations up to 10 mM act as inhibitors. Likewise, the stability of the industrial enzyme in the presence of organic solvents and detergents are the most noticeable features for their diverse applications. In the present study, Pectate lyase activity was inhibited by adding SDS, EDTA Tween 20, and mercaptoethanol. Only Tween-20 has a significant impact on enzyme activity at higher concentrations. This could be due to changes in the secondary and tertiary conformation of proteins which alters their conformation and makes the enzyme more conformational suitable for catalysis. Similarly, an organic solvent such as methanol chloroform stimulates the enzyme activity, while acetone and isopropanol have almost negligible impact on the enzyme activity. Similar results for stimulation of pectinase activity in the presence of EDTA, Trixton-100, Tween-20, and Tween-80 were obtained with the pectinase from *Bacillus* sp*.*^35,44,45^.

The experiment was initiated with only single carbon sources as previous results on single-factor analysis showed that including any other carbon sources leads to the catabolic repression, and microbe's utilized readily available simple carbon sources than complex^46^. The results obtained through the present experiment confirm that pectin is the sole requirement as a carbon source and need most for the pectate lyase production. Several researchers obtained similar results with a higher concentration of pectin in the media^24,47,48^. The carbon source (NH_4_)_2_SO_4_ and initial pH were the most important factors in developing PGL and pectin lyase during submerged fermentation^49^. It also stated that the C:N ratio and pH were the most important factors influencing pectate lyase development^50^, and that lactose, tryptone, and (NH_4_)_2_SO_4_ had a significant impact on pectate lyase activity^11^. Nitrogen is the most important element required for synthesizing various biomolecules. The addition of optimum and suitable Nitrogen supplements results in the higher biomass with significant protein production.

Moreover, among the nitrogen supplements viz. NaNO_3,_ ammonium sulphate (NH_4_SO_4_), tryptone, only ammonium sulphate (NH_4_SO_4_) at 0.3 gm% increased pectate lyase production to 1.0 U/ml. Oumer and Abate obtained similar results, and Sarvamangala^35^ and Dayanand^51^ stated that ammonium sulfate (NH_4_SO_4_) and ammonium nitrate (NH_4_NO_3_) increased pectinase productivity under solid-state fermentation.

The most important aspects of any industry are the cost–benefit ratio. The bacterial enzymes are cheap sources for the clarification purpose with ease of availability and purification. The apple juice is the second most utilized fruit juice^52^. The recovery of the juice, reduction in turbidity, increase in the total soluble solids and antioxidants after enzymatic treatments would be on the positive side compared to mechanical methods of juice clarification while keeping color, flavor, and nutritional properties. The pectin polymer present in the raw juices makes the juice viscous and fussy, which is unlikely from the consumer's point of view. The present experiment proved to help reduce the turbidity by increasing the clarity by 60.00% to 49.91% for different juices with one hour of incubation. These clarity percentages would also be helpful during the filtration process.

Swain et al^53^ reported the yield of carrot juice with better clarity was obtained while increasing incubation time till 8 h. Bhardwaj and Garg^54^ reported that 2 h incubation gave 45.3 and 49.9% carrot juice yield. The other study with little different aspects of enzyme concentration was also supported to our results as a higher concentration of enzyme exhibited better juice clarification^55^. Demir^56^ studied alkaline Pectate lyase of *Brevibacillus borstelensis* (P35) for their application for juice clarification and inferred that 2 h incubation was enough to get clear juice.

## Conclusion

Pectate lyase of bacterial origin is gaining industrial signiuficance over the fungal pectinases as they are produced in relatively shorter incunation period, with less viscocity, and exhibit more activity than fungal pectinase. Pectinases are frequently employed to clarify fruit juices under alkaline and high-temperature conditions. *Bacillus* spp. has a great potential to produce pectinases. However, industrial application demands a thermostable and alkali stable enzyme that is not affected by the inhibitors. The present experiment reported pectate lyase production from *Bacillus subtilis* PKC2, *Bacillus licheniformis* PKC4, *Paenibacillus lactis* PKC5, and *Bacillus sonorensis* ADCN. We report *Paenibacillus lactis* PKC5 as a potent producer of pectate lyase. This isolate produced maximum enzyme in a shorter incubation period of 48 h compared to 54–72 h of incubation required by other organisms. The enzyme was purified with 80% ammonium sulphate, giving a good enzyme yield with higher activity. SDS-PAGE revealed 44 kDa molecular weight of purified enzyme. The purified enzyme exhibited thermostability up to 50 °C temperature and stability at 8.0 pH. The 5 mM concentration of metal ions and 25% (v/v) organic solvents significantly enhanced the enzyme activity. While detergent showed inhibitory effects. The media optimization with a higher concentration of carbon sources with a lower protein concentration was found to be optimal for maximum enzyme production. Earlier work suggested using various media components for optimum production of the enzyme. At the same time, the present study reports pectin and ammonium sulfate as the significant components for the higher yield of the enzyme, thus reducing the cost of enzyme production. The *K*m (8.90 mg/ml) and *V*max (4.578 μmol/ml/min) showed better substrate affinity. The purified enzyme resulted in significant clarification of juices; for grape juice, apple juice, and orange juice, the clarification was 60.37%, 59.36%, and 49.91%, respectively. The isolated pectate lyase clarified the juice up to 60% in an h of incubation, vis-à-vis 50% juice clarification obtained in 2–8 h incubation as reported by others.

## Submission declaration and verification

Submission of an article implies that the work described has not been published previously in any form.

## Supplementary Information

Supplementary Information.

## References

[1] Gummadi SN, Panda T (2003). Purification and biochemical properties of microbial pectinases—a review. Process Biochem..

[2] Ramakanth, N.V., Anuradha, K., & Naga Padma, P. Alkaline polygalacturonases from thermotolerant pectinolytic bacteria from diverse sources. *Int. J. Sci. Res. Publ.* **4,** 2250–3153 (2014). www.ijsrp.org.

[3] Taper AR, Jain RK (2014). Pectinases: enzymes for fruit processing industry. Int. Food Res. J..

[4] Sun L, van Nocker S (2010). Analysis of promoter activity of members of the PECTATE LYASE-LIKE (PLL) gene family in cell separation in Arabidopsis. BMC Plant Biol..

[5] Tariq A, Latif Z (2012). Isolation and biochemical characterization of bacterial isolates producing different levels of polygalacturonases from various sources. Afr. J. Microbiol. Res..

[6] Kashyap DR, Vohra PK, Chopra S, Tewari R (2001). Applications of pectinases in the commercial sector: a review. Bioresour. Technol..

[7] Jayani RS, Saxena S, Gupta R (2005). Microbial pectinolytic enzymes: a review. Process Biochem..

[8] Kavuthodi B, Thomas S, Sebastian D (2015). Co-production of pectinase and biosurfactant by the newly isolated strain bacillus subtilis BKDS1. Br. Microbiol. Res. J..

[9] Aaisha GA, Barate DL (2016). Pectinolytic bacteria #only primary screening #. Int. J. Curr. Microbiol. Appl. Sci..

[10] Mercimek Takcı HA, Turkmen FU (2016). Extracellular pectinase production and purification from a newly isolated bacillus subtilis strain. Int. J. Food Prop..

[11] Yu P, Xu C (2018). Production optimization, purification and characterization of a heat-tolerant acidic pectinase from *Bacillus* sp. ZJ1407. Int. J. Biol. Macromol..

[12] Lee WC, Yusof S, Hamid NSA, Bahrain BS (2006). Optimizing conditions for enzymatic clarification of banana juice using response surface methodology (RSM). J. Food Eng..

[13] Sandri IG, Fontana RC, Barfknecht DM, da Silveira MM (2011). Clarification of fruit juices by fungal pectinases. LWT Food Sci. Technol..

[14] Sharma DC, Satyanarayana T (2012). Biotechnological potential of agro residues for economical production of thermoalkali-stable pectinase by bacillus pumilus dcsr1 by solid-state fermentation and its efficacy in the treatment of ramie fibres. Enzyme Res..

[15] Ahlawat S, Mandhan RP, Dhiman SS, Kumar R, Sharma J (2008). Potential application of alkaline pectinase from Bacillus subtilis SS in pulp and paper industry. Appl. Biochem. Biotechnol..

[16] Murthy PS, Madhava Naidu M (2011). Improvement of robusta coffee fermentation with microbial enzymes. Eur. J. Appl. Sci..

[17] Najafian L, Ghodsvali A, Haddad Khodaparast MH, Diosady LL (2009). Aqueous extraction of virgin olive oil using industrial enzymes. Food Res. Int..

[18] Yu P, Zhang Y, Gu D (2017). Production optimization of a heat-tolerant alkaline pectinase from Bacillus subtilis ZGL14 and its purification and characterization. Bioengineered.

[19] Suneetha, V., & Prathyusha, K. Bacterial pectinases and their potent biotechnological application in fruit processing/juice production industry: a review. *J. Phytol.* **3**: 16–19 (2011). http://journal-phytology.com/index.php/phyto/article/viewArticle/7261.

[20] Kumar S, Stecher G, Tamura K (2016). MEGA7: molecular evolutionary genetics analysis version 7.0 for bigger datasets. Mol. Biol. Evol..

[21] Miller GL (1959). Use of dinitrosalicylic acid reagent for determination of reducing sugar. Anal. Chem..

[22] Lowry OH, Rosebrough NJ, Farr AL, Randall RJ (1951). Protein measurement with the Folin phenol reagent. J. Biol. Chem..

[23] Plackett RL, Burman JP (1946). The design of optimum multifactorial experiments. Biometrika.

[24] Roy K, Dey S, Uddin MK, Barua R, Hossain MT (2018). Extracellular pectinase from a novel bacterium chryseobacterium indologenes strain SD and its application in fruit juice clarification. Enzyme Res..

[25] Nannipieri P, Ascher J, Ceccherini MT, Landi L, Pietramellara G, Renella G, Valori F (2007). Microbial diversity and microbial activity in the rhizosphere. Cienc. Del Suelo..

[26] Burns RG (1982). Enzyme activity in soil: location and a possible role in microbial ecology. Soil Biol. Biochem..

[27] Tanabe H, Yoshihara K, Tamura K, Kobayashi Y, Akamatsu I, Niyomwan N, Footrakul P (1987). Pretreatment of pectic wastewater from orange canning process by an alkalophilic *Bacillus* sp. J. Ferment. Technol..

[28] Pedrolli DB, Monteiro AC, Gomes E, Carmona EC (2009). Pectin and pectinases: production, characterization and industrial application of microbial pectinolytic enzymes. Open Biotechnol. J..

[29] Kavuthodi B, Sebastian D (2018). Review on bacterial production of alkaline pectinase with special emphasis on *Bacillus* species. Biosci. Biotechnol. Res. Commun..

[30] Manal SS, Sahar SM, Manal GM, Mohsen MA, Osama HES (2016). purification, and kinetics of pectinase production from Paenibacillus lactis NRC1 locally isolated from Egyptian mangrove habitat. Der. Pharma. Chem..

[31] Akinyemi B, Buraimoh O, Ogunrinde O, Amund O (2017). Pectinase production by *Bacillus* and *Paenibacillus* sp. isolated from decomposing wood residues in the lagos lagoon. J. Trop. Life Sci..

[32] Giacobbe, S., Pepe, O., Ventorino, V., Birolo, L., Vinciguerra, R., & Faraco, V. Enzyme from *Paenibacillus xylanolyticus*, **9**: 4873–4887 (2014)

[33] Mohamed Helal El-Sayed IAEE (2017). *Paenibacillus* sp. strain NBR–10, a thermophilic soil-isolated bacterium with thermo-alkali stable pectinase activity. J. Appl. Environ. Biol. Sci..

[34] Torimiro N, Okonji ER (2013). A comparative study of pectinolytic enzyme production by Bacillus species. Afr. J. Biotechnol..

[35] Oumer OJ, Abate D (2018). Comparative studies of pectinase production by Bacillus subtilis strain Btk 27 in submerged and solid-state fermentation. Biomed. Res. Int..

[36] Nawawi MH, Mohamad R, Tahir PM, Saad WZ (2017). Extracellular xylanopectinolytic enzymes by Bacillus subtilis ADI1 from EFB's compost. Int. Sch. Res. Not..

[37] Remoroza C, Wagenknecht M, Buchholt HC, Moerschbacher BM, Gruppen H, Schols HA (2015). Mode of action of Bacillus licheniformis pectin methylesterase on highly methylesterified and acetylated pectins. Carbohydr. Polym..

[38] Mukhopadhyay A, Dasgupta AK, Chattopadhyay D, Chakrabarti K (2012). Improvement of thermostability and activity of pectate lyase in the presence of hydroxyapatite nanoparticles. Bioresour. Technol..

[39] Ouattara HG, Reverchon S, Niamke SL, Nasser W (2010). Biochemical properties of pectate lyases produced by three different bacillus strains isolated from fermenting cocoa beans and characterization of their cloned genes†. Appl. Environ. Microbiol..

[40] Gophanea SR, Khobragadea CN, Jayebhayea SG (2016). Extracellular pectinase activity from Bacillus Cereus GC subgroup a: isolation, production, optimization and partial characterisation. J. Microbiol. Biotechnol. Food Sci..

[41] Khatri, B.P., Bhattarai, T., Shrestha, S., & Maharjan, J. Alkaline thermostable pectinase enzyme from *Aspergillus niger* strain MCAS2 isolated from Manaslu Conservation Area, Gorkha, Nepal, Springerplus. 4 (2015). doi:10.1186/s40064-015-1286-y. 10.1186/s40064-015-1286-y PMC4564381 26380164

[42] Sunnotel O, Nigam P (2002). Pectinolytic activity of bacteria isolated from soil and two fungal strains during submerged fermentation. World J. Microbiol. Biotechnol..

[43] Anggraini, D.P., Sulistiana, D., Agustina, D.K., & Ulimaz, A. Determination of kinetic parameters and the effect of ion Mg^2+^ inhibition into pectinase activities. *J. Penelit. Dan Pengkaj. Ilmu Pendidik. e-Saintika*. 4 (2020) 112. doi:10.36312/e-saintika.v4i2.170.

[44] Li Z, Bai Z, Zhang B, Li B, Jin B, Zhang M, Lin F, Zhang H (2012). Purification and characterization of alkaline pectin lyase from a newly isolated *Bacillus clausii* and its application in elicitation of plant disease resistance. Appl. Biochem. Biotechnol..

[45] Amid M, Manap Y, Zohdi K (2014). Purification and characterization of thermo-alkaline pectinase enzyme from *Hylocereus polyrhizus*. Eur. Food Res. Technol..

[46] Ahlawat S, Dhiman SS, Battan B, Mandhan RP, Sharma J (2009). Pectinase production by Bacillus subtilis and its potential application in bio preparation of cotton and micropoly fabric. Process Biochem..

[47] Fawole, S.A., & Odunfa, O.B. Pectolytic moulds in Nigeria, 15: 266–268 (1992). doi:10.1111/j.1472-765X.1992.tb00780.x.

[48] Mohandas A, Raveendran S, Parameswaran B, Abraham A, Athira RSR, Mathew AK, Pandey A (2018). Production of pectinase from bacillus sonorensis MPTD1. Food Technol. Biotechnol..

[49] Guo, F., Li, X., Zhao, J., Li, G., Gao, P., & Han, X. Optimizing culture conditions by statistical approach to enhance production of pectinase from Bacillus sp. Y1, (2019). doi:10.1155/2019/8146948. 10.1155/2019/8146948 PMC6402201 30915361

[50] Sharma DC, Satyanarayana T (2006). A marked enhancement in the production of a highly alkaline and thermostable pectinase by Bacillus pumilus dcsr1 in submerged fermentation by using statistical methods. Bioresour. Technol..

[51] Patil SR, Dayanand A (2006). Exploration of regional agrowastes for pectinase production by *Aspergillus niger*. Food Technol. Biotechnol..

[52] Kahle K, Kraus M, Richling E (2005). Polyphenol profiles of apple juices. Mol. Nutr. Food Res..

[53] Swain MR, Ray RC (2010). Production, characterization and application of a thermostable exo-polygalacturonase by Bacillus subtilis CM5. Food Biotechnol..

[54] Bhardwaj V, Garg N (2014). Production, purification of pectinase from *Bacillus* sp. MBRL576 isolate and its application in extraction of juice. Int. J. Sci. Res..

[55] Joshi VK, Parmar M, Rana N (2011). Purification and characterization of pectinase produced from apple pomace and evaluation of its efficacy in fruit juice extraction and clarification. Indian J. Nat. Prod. Resour..

[56] Demir N, Nadaroglu H, Demir Y, Isik C, Taskin E, Adiguzel A, Gulluce M (2014). Purification and characterization of an alkaline pectin lyase produced by a newly isolated brevibacillus borstelensis (p35) and its applications in fruit juice and oil extraction. Eur. Food Res. Technol..

