# Supplementary material for: Production, statistical optimization, and functional characterization of alkali stable pectate lyase of Paenibacillus lactis PKC5 for use in juice clarification
Source: Sci Rep. 2022 May 9;12:7564. doi: 10.1038/s41598-022-11022-0 (PMC9085886; doi:10.1038/s41598-022-11022-0)
Supplement: Supplementary file 1 — Supplementary Information. [file 41598_2022_11022_MOESM1_ESM.pdf]

Production, Statistical Optimization, and Functional Characterization of Alkali Stable Pectate Lyase of *Paenibacillus lactis* PKC5 for use in Juice Clarification

Priyanka Sheladiya[1], Chintan Kapadia[1]*, Vimal Prajapati[2], Hesham Ali El Enshasy[3,4,5], Roslinda Abd Malek[3], Najat Marraiki[6], Nouf S. S. Zaghloul[7] and R. Z. Sayyed[8]*

[1] Department of Plant Molecular Biology and Biotechnology, ASPEE College of Horticulture and Forestry, Navsari Agricultural University, Navsari, 396450, India; sheladiyapriyanka1122@gmail.com (P.S.); chintan_bt@yahoo.co.in (C.K.);

[2] ASPEE SHAKILAM Biotechnology Institute; Navsari Agricultural University; Ghod Dod Road, Athwa Farm, Surat-395 007, India; vimalprajapati@nau.in

[3] Institute of Bioproduct Development, Universiti Teknologi Malaysia (UTM), Skudai 81310, Johor Bahru, Malaysia. henshasy@ibd.utm.my, roslinda@ibd.utm.my

[4] School of Chemical and Energy Engineering, Faculty of Engineering, Universiti Teknologi Malaysia (UTM), Skudai 81310, Johor Bahru, Malaysia.

[5] City of Scientific Research and Technology Applications, New Burg Al Arab 21934, Alexandria, Egypt.

[6] Department of Botany and Microbiology, College of Science, King Saud University, P.O. 8455, Riyadh, 11451, Saudi Arabia; najat@ksu.edu.sa

[7] Bristol Centre for Functional Nanomaterials, HH Wills Physics Laboratory, Tyndall Avenue, University of Bristol, Bristol, BS8 1FD, UK; nouf.zaghloul@bristol.ac.uk

[8] Department of Microbiology, PSGVP Mandal's Shri S I Patil Arts, G B Patel Science, and STKVS Commerce College, Shahada 425409, India

* Corresponding author: sayyedrz@gmail.com; chintan_bt@yahoo.co.in

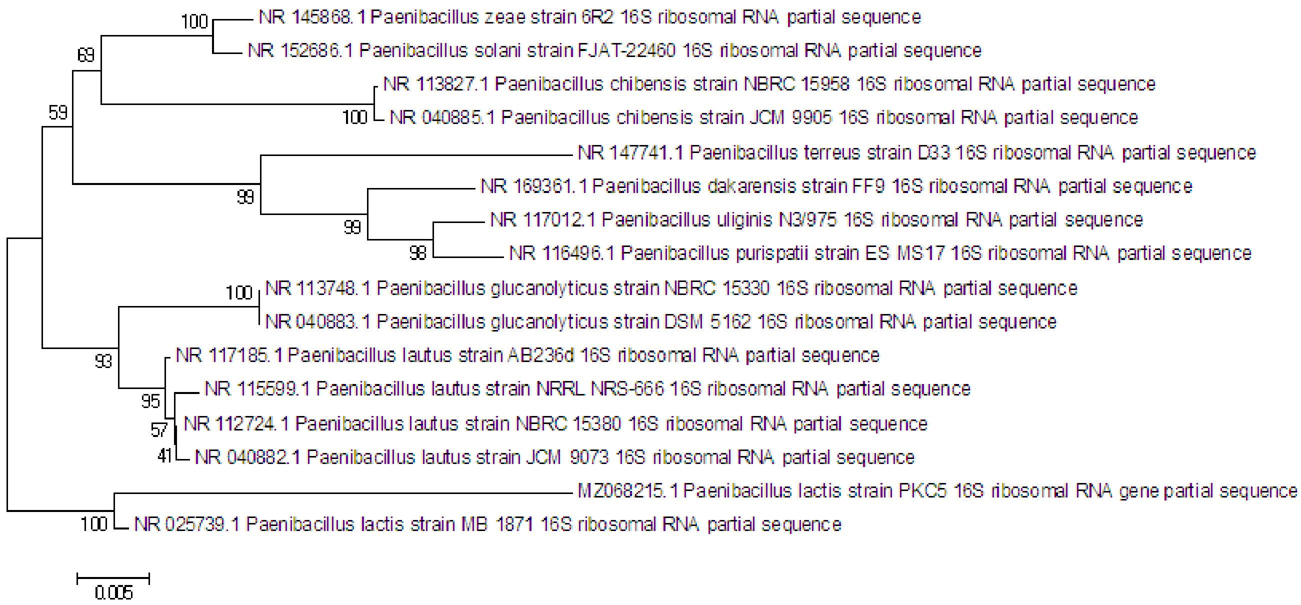

Figure S1a. Phylogenetic tree formed based on the 16S rRNA gene sequences of *Paenibacillus lactis strain PKC5* with other *Paenibacillus* sp. obtained from GenBank database. The accession number has been represented before their name in the tree.

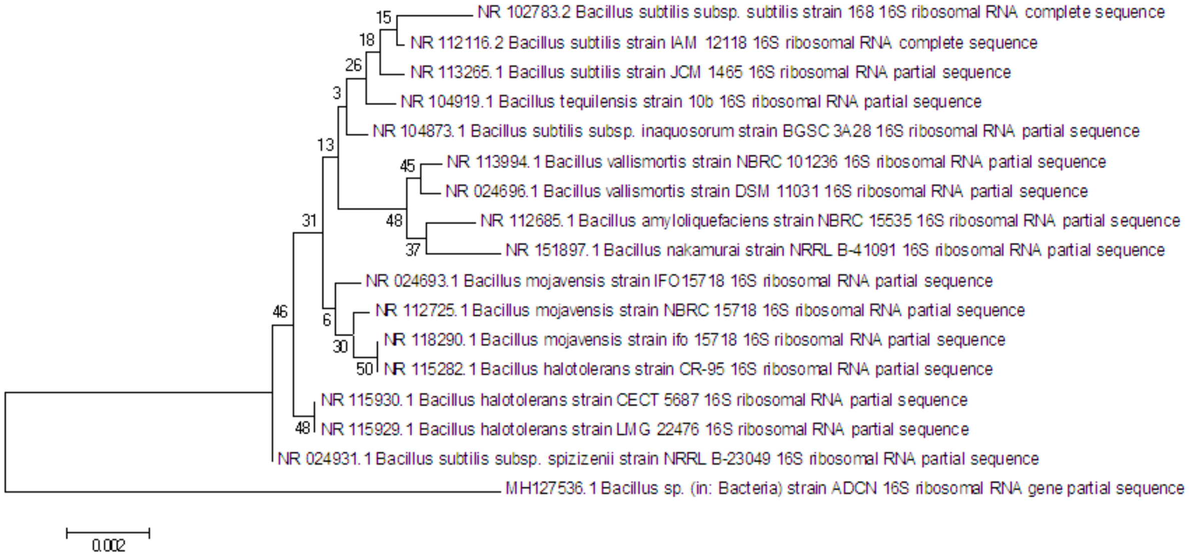

Figure S1b. Phylogenetic tree formed based on the 16S rRNA gene sequences of *Bacillus sp.* with another *Bacillus* sp. obtained from GenBank database. The accession number has been represented before their name in the tree.

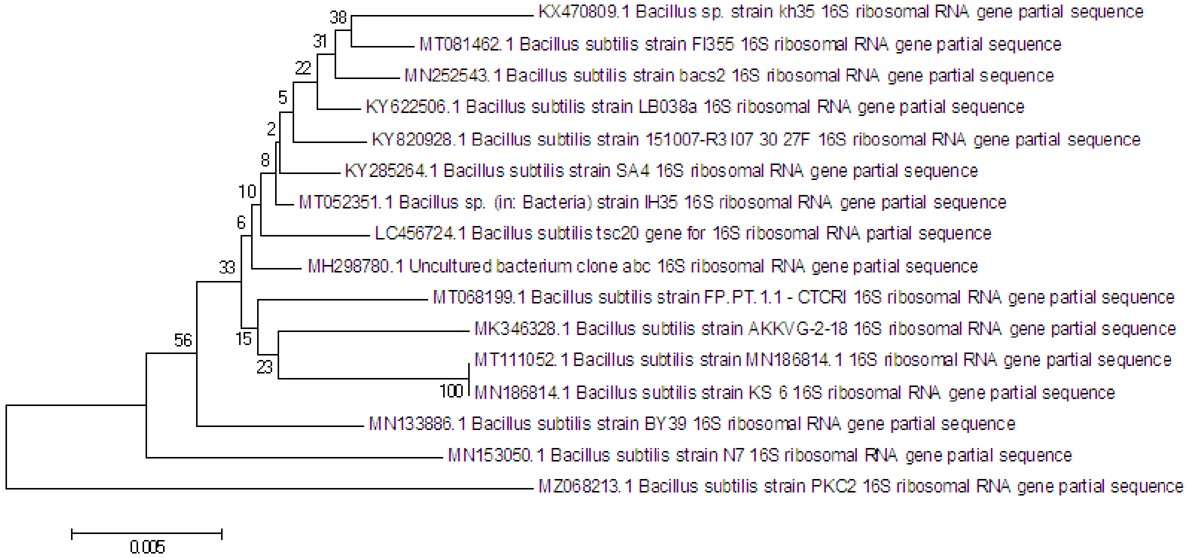

Figure S1c. Phylogenetic tree formed based on the 16S rRNA gene sequences of *Bacillus Subtilis* strain P*KC2* with other *Bacillus* sp. obtained from GenBank database. The accession number has been represented before their name in the tree.

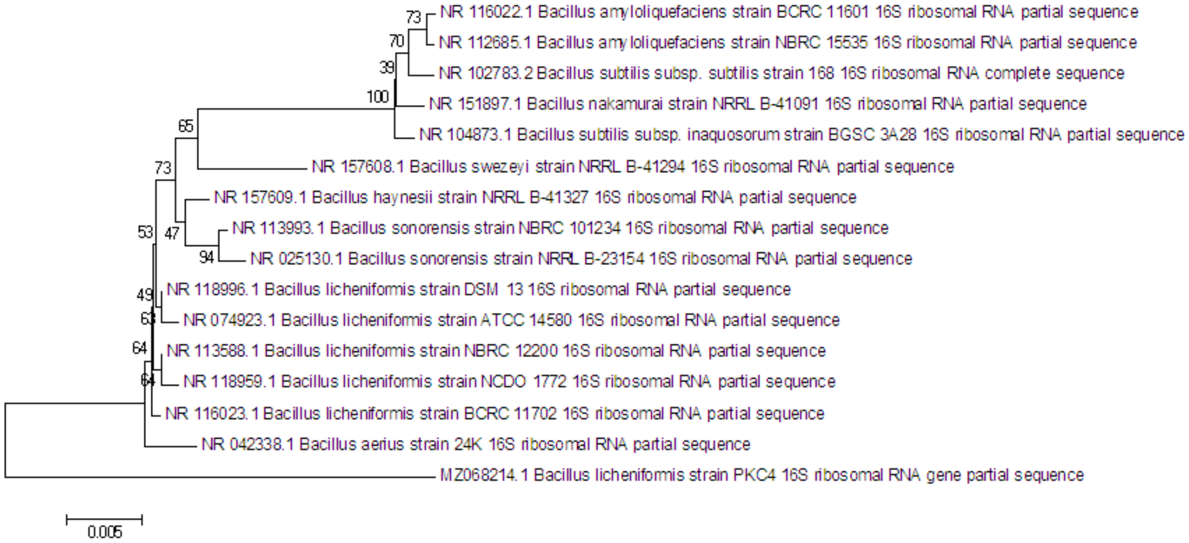

Figure S1d. Phylogenetic tree formed based on the 16S rRNA gene sequences of *Bacillus Licheniformis strain PKC4* with other *Bacillus Licheniformis* obtained from GenBank database. The accession number has been represented before their name in the tree.
